# Effects of Acute Ethanol Intoxication on Spinal Cord Injury Outcomes in Female Mice

**DOI:** 10.1089/neu.2023.0077

**Published:** 2023-11-30

**Authors:** Ethan P. Glaser, Andrew N. Stewart, Julia E. Jagielo-Miller, Caleb S. Bailey, Mark A. Prendergast, John C. Gensel

**Affiliations:** ^1^ Department of Physiology, Spinal Cord and Brain Injury Research Center, College of Medicine, University of Kentucky, Lexington, Kentucky, USA.; ^2^ Department of Psychology, University of Kentucky, Lexington, Kentucky, USA.

**Keywords:** alcohol, anesthesia, ethanol metabolism, neuropathology

## Abstract

Approximately one in three traumatic spinal cord injuries (SCIs) occurs during or shortly after the consumption of alcohol. A small number of retrospective clinical studies report variable effects of alcohol intoxication on mortality, neurological recovery, and complications after SCI. Some of these studies demonstrate a protective effect of alcohol intoxication on SCI outcomes, whereas others show an increased complication risk. Pre-clinical studies in rat, ferret, and feline SCI models report a detrimental effect of ethanol intoxication on hemorrhage, motor recovery, and biochemical markers of tissue injury. However, no studies to date have investigated the neuropathological consequences of ethanol intoxication at the time of SCI or the reciprocal effect of SCI on ethanol metabolism. Therefore, we combined a pre-clinical mouse model of acute ethanol intoxication and experimental vertebral level T9 contusion SCI to investigate their interactive effects in female mice. We first investigated the effect of SCI on ethanol metabolism and found that T9 SCI does not alter ethanol metabolism. However, we did find that isoflurane anesthesia significantly slowed ethanol metabolism independent of SCI. We also determined how acute ethanol intoxication at the time of SCI alters locomotor recovery and lesion pathology. Using the Basso Mouse Scale (BMS) and CatWalk XT Gait Analysis System, we assessed locomotor recovery for 6 weeks after injury and observed that acute ethanol intoxication at the time of injury did not alter locomotor recovery. We also found no effect of ethanol intoxication on heat hyperalgesia development. There was, however, a detrimental effect of ethanol on tissue sparing after SCI. Therefore, we conclude that acute alcohol intoxication at the time of injury may contribute to the neuropathological consequences of SCI.

## Introduction

Twenty to forty-seven percent of individuals with spinal cord injury (SCI) are reported to have had an elevated blood alcohol content (BAC) at the time of their injury.^1–3^ Although acute ethanol intoxication is found in about one-third of SCI cases, its ramifications on clinical outcomes and recovery are not well understood. Clinical studies investigating the effect of an elevated BAC on SCI outcomes have varying results regarding sensorimotor recovery, morbidity, and mortality.^4–6^ A retrospective case review reported that individuals with a BAC above 0.08% on admission have longer hospital and intensive care unit (ICU) stays, and increased risk of complications, but no increased risk of mortality.^4^ Another study reported no effect of BAC on motor and sensory recovery, pain sensitivity, and mortality after SCI.^6^ Most recently, a study demonstrated a protective effect of acute ethanol intoxication on motor recovery with no effect on recovery of sensory light touch and sensory proprioception.^5^ Pre-clinical studies done in rat, feline, and ferret SCI models demonstrated that acute ethanol intoxication caused more severe hemorrhage, increased free fatty acids, and worse motor scores.^7–9^ However, tissue pathology and ethanol metabolism after SCI were not measured in these studies. Further, to the best of our knowledge, the effects of elevated BAC at the time of SCI have not been evaluated in mice.

We, therefore, employed a mouse model of “binge-like” acute ethanol intoxication with concomitant contusion SCI to investigate the effects of alcohol on locomotor recovery, hyperalgesia, and tissue pathology. We used a single dose of ethanol to mimic binge-drinking behavior that is reported by spinal cord injured individuals prior to their injury.^10^ In humans, binge drinking is the consumption of 4–5 or more drinks in one sitting.^11^ To model binge drinking, we administered a single 3 g/kg dose. Mice metabolize ethanol 5 times faster than humans and therefore a high initial dose is required to achieve a comparable area under the alcohol concentration versus time curve (AUC).^12^ We found that acute ethanol intoxication did not alter locomotor recovery or heat hyperalgesia after SCI. We did, however, find that ethanol intoxication exacerbated tissue loss and that anesthesia slowed ethanol metabolism.

## Methods

### Animals

All procedures were approved by the University of Kentucky's Institutional Animal Care and Use Committee. All mice were group housed at 3–5 mice per cage under a normal light cycle. Female 3- to 4-month-old C57/BL6J mice (Jackson Laboratories) were used for all experiments.

### Spinal cord injury

For the SCI surgery, anesthesia was induced using 5% isoflurane and mice were maintained at 2–3% during the procedure. Mice received a surgical laminectomy followed by a 75 kdyn vertebral level T9 contusion SCI (Infinite Horizons Impactor; IH) as previously described.^13,14^ Based upon *a priori* exclusion criteria, any mouse receiving an SCI with abnormalities in the force versus time curve generated by the IH device was excluded from analysis. Abnormalities meriting exclusion include bone hits or instability in the spinal cord at the time of injury and occurred in four mice (two from each group). After exclusion criteria were applied, force and displacement were similar between groups (force: vehicle = 76.42 ± 1.83 kdyn, ethanol = 77.17 ± 2.59 kdyn, *p* = 0.421; displacement: vehicle = 724.3 ± 119.7 μm, ethanol = 655.0 ± 99.0 μm, *p* = 0.137). After injury, muscle and skin incisions were closed using monofilament suture. Mice that opened their incision more than once were excluded from the study. One mouse was excluded from the vehicle group for this reason. Mice received an analgesic (Buprenex SR, 1.0 mg/kg) and antibiotic (1646RX, Enrofloxacin, 5.0 mg/kg, Enroflox), as well as saline (1.0 mL/day for 5 days) following surgery. Mice in the ethanol metabolism experiment did not receive analgesic or antibiotic until after the blood was collected to prevent hemodilution. Bladders from all SCI mice were manually expressed twice per day during the study.

### Ethanol administration and plasma ethanol concentration measurements

To achieve a rapid and reproducible plasma ethanol concentration (PEC) of approximately 250–300 mg/dL, ethanol was delivered via intraperitoneal (i.p.) injection 40–50 min prior to SCI; i.p. administration was chosen over oral administration because compared with oral gavage i.p. injections of ethanol are more reproducible, safer for the animal, and cause a higher PEC.^15,16^ A 250–300 mg/dL PEC range was ideal because it has been used previously in rodent models of SCI and it effectively models a binge-drinking episode in rodents.^7,12^ It is important to note that PEC is 1.09 to 1.18 times higher than BAC because serum and plasma samples contain more water than whole blood and alcohol is distributed uniformly throughout body water.^17^

To determine the effect of SCI and isoflurane anesthesia on ethanol metabolism, 14-week-old female mice (ethanol [etoh] group *n* = 8, isoflurane [iso] + etoh group *n* = 11, SCI+iso+etoh group *n* = 12) were given an i.p. injection of 20% (v/v) ethanol in saline at a dose of 3 g/kg. The same dosing paradigm was used to assess functional recovery and tissue pathology. In the cohort of mice used to measure ethanol metabolism each mouse in the iso+etoh group was paired with a mouse from the SCI+iso+etoh group. Both mice were anesthetized for the same length of time and received the isoflurane dose via a dual output nose-cone system. Blood was taken approximately every hour after dosing and only one sample was taken from each mouse. Blood was collected from the facial vein into heparin-coated capillary tubes (16.440.100, Sarstedt) and transferred into 1.5 mL ethylenediaminetetraacetic acid (EDTA)-coated tubes. Plasma was separated from whole blood via centrifugation for 10 min at 1500*g* at room temperature. Plasma was isolated and stored at −80°C until plasma ethanol concentration was measured using the Analox AM1 Analyser (Analox Instruments). The Analox AM1 Analyser uses an alcohol oxidase enzyme-based detection assay to quantify ethanol concentration in a sample. The alcohol oxidase enzyme catalyzes the reaction of ethanol and oxygen into acetaldehyde and hydrogen peroxide. The instrument measures the rate of oxygen consumption, which is directly proportional to sample ethanol concentration. All samples were run in triplicate and an average of the three values were used as the PEC.

### Behavioral analysis

Locomotor assessments were made using the CatWalk XT Gait Analysis System (Noldus) and the Basso Mouse Scale (BMS).^18^ For CatWalk analysis, mice underwent three testing sessions: 1 week prior to injury and 4 and 6 weeks post-injury (wpi). The CatWalk features a red overhead light and green illuminated walkway that reflects light in response to the contact of the mouse's paw that is then captured via calibrated video recordings. Gait analysis was performed by the same researcher in a dark room. Animals were acclimated in the room for 30 min prior to testing. For a single run, the mouse was first placed in the open end of the CatWalk under the red ceiling light and allowed to walk across the walkway to the darkened escape enclosure. Each mouse completed three continuous runs on each analysis day and a minimum of three valid runs, or complete walkway crossings, were obtained for each subject. Trials in which the mouse stopped or turned around during a run were excluded from analysis. Mice that did not complete three continuous runs after 25 attempts were excluded from analysis.

The BMS utilizes a 9-point rating scale to characterize gross locomotor functions ranging from complete paralysis (score 0) to normal functions (score 9) as mice explore an open field for 4 min.^18^ BMS scores were obtained at 1, 3, 7, 14, 21, 28, 35, and 42 days post-injury (dpi) by two experimental raters who were blinded to treatment groups. In the week prior to injury animals were acclimated to the sandbox used for BMS scoring once for 5 min. Each hindlimb was scored separately based on movement (e.g., ankle placement and stepping), coordination, and trunk stability. Averaging both hindlimb scores generated a single score for each mouse. BMS sub-scores were derived from observations made during BMS scoring. BMS sub-scores are based on features of locomotion such as trunk stability, coordination, and paw placement that are observable in higher-functioning mice.^18^ BMS sub-scores permitted a better resolution to differentiate between mice with higher BMS scores.

### Hargreaves thermal sensitivity assessment

The Hargreaves test of thermal hypersensitivity was used as previously described^19^ and modified for mice^20,21^ (37370, Ugo Basile, Germany). Mice were acclimated to testing boxes (37370, Ugo Basile, Germany) for 2 h/day for 2 days prior to testing and acclimated for 1 h prior to data acquisition. Testing was performed pre-SCI and at 42 dpi. For each test, an infrared laser was aimed at the plantar surface of each hindpaw. The laser is calibrated to reach a maximum temperature of 55°C over a 25-sec period. The time at which the animal responded to the thermal stimulus was recorded. Responses were only counted if they were a deliberate flinch, indicated by an abrupt lifting and replacement of the paw, sometimes followed by licking of the paw. Responses in which mice moved without an explicit reaction to the heat were not considered valid. Each mouse's paws were given at least 1 min of rest in between trials. Many mice were tested simultaneously, which enabled the experimental tester to switch between mice regularly. Three response times were obtained per hindfoot and the six recorded times for both feet were averaged to generate one score per mouse per time-point.

### Immunohistochemistry

At 42 dpi mice were anesthetized using an overdose of ketamine (4.0–5.0 mg) and xylazine (0.4–0.5 mg) and euthanized via transcardial perfusion using phosphate buffered saline (PBS) followed by 4% formaldehyde (Millipore Sigma). Spinal cords were extracted and post-fixed in 4% formaldehyde for 2 h at room temperature before being transferred to 0.1 M phosphate buffer (PB) overnight. Spinal cords were dehydrated in 30% sucrose for a week and embedded in optimal cutting temperature (OCT) compound (4583, Sakura Finetek USA, Inc.). Five spinal cords were embedded in each OCT block with 2–3 cords per group randomly selected. Serial 10 μm coronal sections were collected on ColorFrost Plus Microscope Slides (22-037-246, Fisher Scientific). Ten sets of tissue were generated that spanned the length of the lesion and the distance between each section on a single slide was 100 μm.

All slide-mounted sections received antigen retrieval at 80°C in sodium citrate buffer with 0.1% Tween-20 (pH 6.0) for 5 min. Sections were then treated with 0.3% H_2_O_2_ in 40% methanol and PBS for 30 min to quench endogenous peroxidase activity. Next, sections were blocked in 5% normal goat serum in PBS/0.1% Triton-X 100 for 1 h at room temperature. Sections were then stained overnight at room temperature with neurofilament-200 kD (1:1500; Ck x NF200; NFH, Aves Labs). Sections were washed twice in PBS followed by 1-h long incubation with biotinylated secondary antibody (1:500; Goat anti-chicken, BA9010, Vector Laboratories). Sections were then incubated in avidin biotin complex solution (ABC; 1:200; PK-6100, Vector Laboratories) and developed using 3,3’-diaminobenzidine (DAB; SK-4100, Vector Laboratories). Sections were counter-stained with eriochrome cyanine to visualize spared white matter. Stained slides were dehydrated using graded ethanol dilutions, cleared using Histoclear (101412-878, VWR Scientific), and coverslipped using Permount (SP15-500, Fisher Scientific). Slides were imaged using Axioscan (model Z1, Carl Zeiss AG, Oberkochen, GE) at 20 × magnification and visualized and quantified using Halo software (Indica Labs, Albuquerque, NM, USA).

Tissue sparing was assessed by tracing intact white and gray matter at 100 μm intervals throughout a 1.4 mm span of the lesion centered on the lesion epicenter. The lesion epicenter was quantitatively defined as the section containing the least amount of spared tissue. All analyzed sections were oriented with respect to the lesion epicenter. Lesion length was defined as the total span of the spinal cord in which at least 5% of the cord area was negative for both myelin and axons (i.e., 5% damaged).

### Exclusion criteria summary

Sixteen mice were originally included in each group. Of the 16 original mice in the vehicle group: 4 mice were excluded because of abnormalities in the force × displacement curve during injury and 1 mouse was excluded for reopening its sutures multiple times; *n* = 11 from the vehicle group for behavioral evaluation. Of the 16 mice in the ethanol group, 2 were excluded for abnormalities in the force × displacement, 1 did not survive the surgery, and 2 were euthanized for extremely poor recovery after SCI at 1 dpi; *n* = 10 from the ethanol group for behavioral evaluation. During analyses of tissue pathology, one block of 5 mice was excluded due to excessive tissue loss due to spinal cord sections falling off the slides. This block included 2 previously excluded subjects and 2 additional mice from the vehicle group and 1 from the ethanol group. Final numbers for anatomical analyses: *n* = 9 for vehicle and *n* = 9 for ethanol group.

### Statistical analysis

All statistical tests were performed using GraphPad Prism software (v9.4.1, Boston, MA, USA). Simple linear regression was used to quantify ethanol metabolism rate. Metabolism rates were compared using analysis of covariance (ANCOVA). AUC comparisons were made using a one-way analysis of variance (ANOVA) with Holm-Sidak post hoc tests. BMS scores, sub-scores, and regularity index scores between treatment groups and over time were compared using a two-way repeated measures ANOVA also with Holm-Sidak post hoc tests. Paw withdrawal latency, stride length, print area, and regularity index comparisons were made with a two-way ANOVA. The lesion area at the epicenter and lesion length were compared using a Student's *t*-test. The lesion area over the length of the lesion was compared using a two-way repeated measures ANOVA with Fischer's post hoc tests. *P* ≤ 0.05 was considered significant.

## Results

**FIG. 1.**
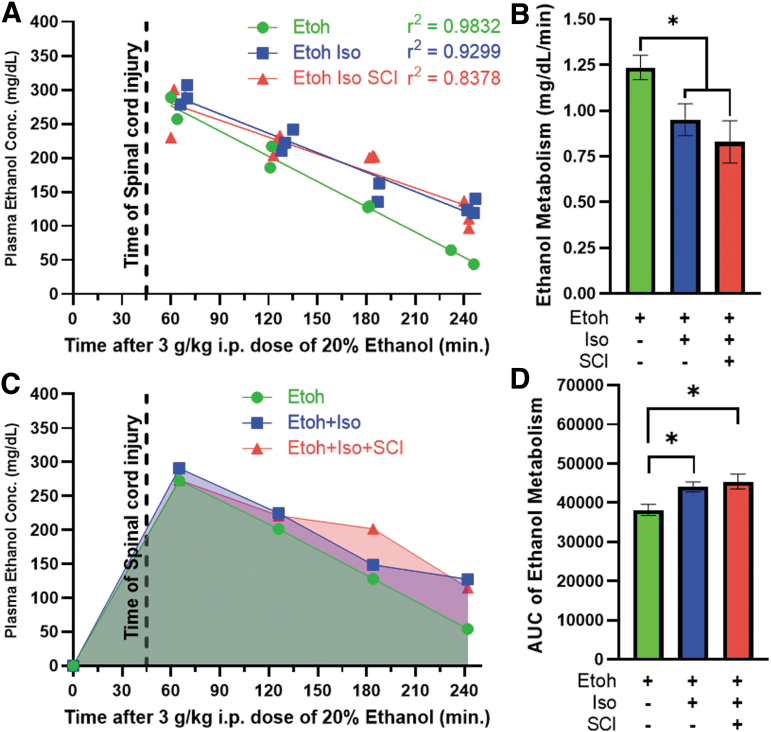
Isoflurane anesthesia, but not SCI, slows ethanol metabolism. **(A)** PEC measurements acquired every hour for 4 h after a single i.p. administration of ethanol (Etoh; time 0; 3.0 g/kg dose). Mice were injured 45 min post-injection (black dotted line). Colored lines represent a simple linear regression calculated based on PEC over time. **(B)** The slope of the lines in A represented as the rate of ethanol metabolism. Isoflurane (Iso) anesthesia slows the rate of ethanol metabolism significantly (*p < 0.05 for Etoh alone vs. Iso conditions); however, SCI does not further reduce the metabolic rate. **(C)** AUC calculated based on the average PEC from each time-point. The black dotted line represents the time of SCI after ethanol administration. AUC represents the total ethanol exposure over time. Mice that were only under anesthesia and mice that were also injured had significantly increased ethanol exposure over the 4-h period (*p* = 0.051, etoh+iso; *p* = 0.019, etoh+iso+SCI; etoh: *n* = 8, etoh+iso: *n* = 11, etoh+iso+SCI: *n* = 12/group). Assessments used include a simple linear regression analysis (A), ANCOVA (B), and one-way ANOVA (C); *p < 0.05 Tukey post hoc, error bars represent SEM (A and C) or SD (B). ANCOVA, analysis of covariance; ANOVA, analysis of variance; AUC, area under the curve; i.p., intraperitoneal; PEC, plasma ethanol concentration; SCI, spinal cord injury; SD, standard deviation; SEM, standard error of the mean.

Our current work aimed to determine the extent to which ethanol intoxication at the time of SCI alters long-term functional and anatomical outcomes. To ensure that mice were exposed to a consistent concentration of ethanol at the time of injury, we first measured the maximum PEC and rate of ethanol metabolism following a single i.p. bolus of ethanol. We included three groups of mice: 1) mice given ethanol only, 2) mice given ethanol and put under isoflurane anesthesia, and 3) mice given ethanol, put under isoflurane anesthesia, and given an SCI. Isoflurane anesthesia significantly slowed the rate of ethanol metabolism (F_(1,27)_ = 7.24, *p* = 0.012) without a compounding SCI effect (F_(1,19)_ = 0.694, *p* = 0.415; Fig. 1B). Additionally, both isoflurane (*p* = 0.05) and SCI (*p* = 0.019) significantly increased the AUC relative to group 1 (no isoflurane, no SCI; Fig. 1C,D). The AUC represents the total exposure to ethanol (Fig. 1C,D). These results show that a single bolus i.p. dose of ethanol achieves a constant and reproducible PEC between 45 and 60 min after injection. In our subsequent cohort, we delivered SCI within 45–60 min after ethanol injection to ensure all mice had a similar BAC at the time of SCI.

**FIG. 2.**
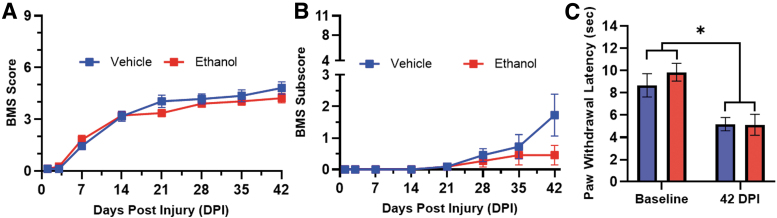
Acute ethanol intoxication at the time of injury does not significantly alter locomotor outcomes or heat hypersensitivity after SCI. Ethanol was administered 45 min prior to SCI and locomotor outcomes were assessed on the BMS **(A)** and BMS sub-score **(B)**. Heat hyperalgesia was evaluated **(C)** prior to SCI (baseline) and 42 DPI after SCI. Ethanol did not alter the degree of hypersensitivity, but significant hypersensitivity was observed after injury relative to baseline in both groups (A–C, Vehicle and Ethanol: *n* = 11/group). Assessments made using two-way repeated measures ANOVA (* = *p* < 0.05). Error bars represent SEM. ANOVA, analysis of variance; BMS, Basso Mouse Scale; DPI, days post-injury; SCI, spinal cord injury; SEM, standard error of the mean.

To test the hypothesis that ethanol exposure at the time of injury alters sensorimotor outcomes we assessed locomotor recovery and the thermal hypersensitivity development for 6 weeks after SCI. Adult (4-month-old) female mice were given ethanol or vehicle (saline) i.p. 45 min prior (vehicle = 53.1 ± 17.9 min, ethanol = 45.3 ± 3.42 min, *p* = 0.163) to receiving a moderate-severe thoracic contusion SCI (75 kdyn IH T9 contusion). Injury force and spinal cord displacement were not significantly different between treatment groups, ensuring that both groups received comparable injuries (force: vehicle = 76.42 ± 1.83 kdyn, ethanol = 77.17 ± 2.59 kdyn, *p* = 0.421; displacement: vehicle = 724.3 ± 119.7 μm, ethanol = 655.0 ± 99.0 μm, *p* = 0.137). As we have reported previously for a moderate-severe SCI, mice recovered from an initial hindlimb paralysis to hindlimb stepping (BMS score of 4–5) by 6 wpi (Fig. 2A,B).^14^ BMS scoring and sub-scoring did not detect a significant effect of ethanol exposure on locomotor recovery (F_(1, 20)_ = 0.42, *p* = 0.526, BMS score) (F_(1, 20)_ = 1.71, *p* = 0.2061, BMS sub-score; Fig. 2A,B). Regarding sensory outcomes, we observed that SCI mice were hypersensitive to heat stimuli (Hargreaves test) 6 weeks after SCI relative to baseline values (F_(1,20)_ = 20.13, *p* = 0.0002; Fig. 2C). Ethanol exposure at the time of injury did not alter heat hypersensitivity development (F_(1,20)=_ 0.49, *p* = 0.49; Fig. 2C).

**FIG. 3.**
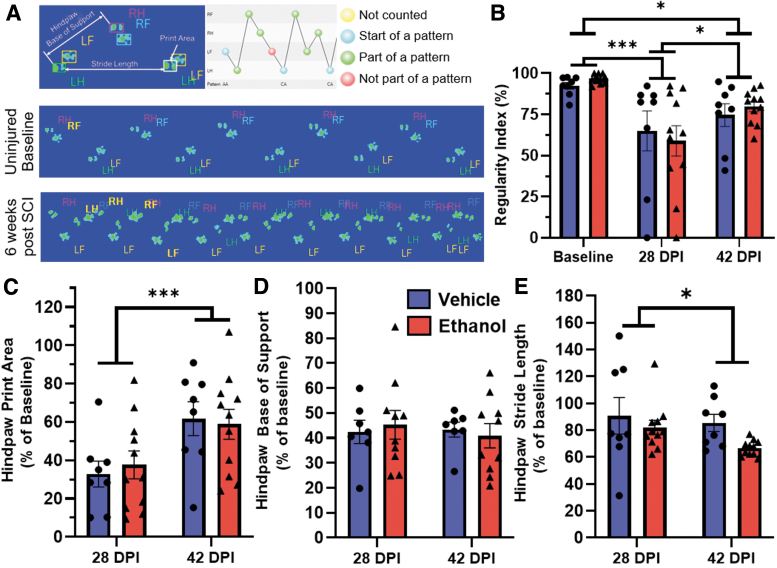
Acute ethanol intoxication at the time of injury does not significantly alter locomotor outcomes after SCI. Ethanol or vehicle was administered 45 min prior to SCI and specific parameters of gait and locomotion were assessed using the CatWalk XT prior to injury and 4 and 6 wpi. An overview of the parameters assessed is shown **(A)**. Representative paw prints are shown (A) for a single mouse before and after SCI. Note the reduced regularity index, base of support, and stride length as well as altered step pattern after SCI. Regularity index **(B)**, hindpaw print area **(C)**, hindpaw base of support **(D)**, and hindpaw stride length **(E)** are shown. Note that for C–E, measures are expressed as a percentage of baseline (100%). Acute ethanol intoxication did not hinder locomotor recovery; however, there was a significant increase in print area between 4 and 6 wpi (C). (Vehicle: *n* = 8, Ethanol: *n* = 11) assessments made using two-way ANOVA (*p < 0.05, **p < 0.01, ***p < 0.001). Error bars represent SEM. DPI, days post-injury; ANOVA, analysis of variance; LF, left front; LH, left hind; RF, right front; RH, right hind; SCI, spinal cord injury; SEM, standard error of the mean; wpi, weeks post-injury.

We also employed the CatWalk XT Gait Analysis System to interrogate more specific parameters of gait and coordination during recovery. We evaluated mice at baseline and at 4 and 6 wpi. Within mouse comparisons before and after injury demonstrate that SCI alters the distances between sequential prints (stride length), reduces print area, causes atypical stepping patterns (regularity index), and decreases the distance between left-right steps (base of support; Fig. 3A). Using the CatWalk XT, we specifically assessed the regularity index, print area, stride length, and base of support of the hindpaws as continuous measures sensitive to moderate-severe SCI (Fig. 3A, bottom). The regularity index measures the percent of step cycles that follow a coordinated step sequence, that is, each paw produces a step for every four-paw sequence^22^ (Fig. 3A, top right). The regularity index was lower than baseline at 4 wpi (*p* < 0.0001), significantly improved at 6 wpi (*p* = 0.017), but still differed from baseline values (*p* = 0.014; F_(2, 34)_ = 14.42, *p* < 0.0001, main effect of time).

There was no difference between treatment groups (F_(1, 17)_ = 0.027, *p* = 0.87, main effect of treatment) (Fig. 3B). Print area is the maximum paw contact area during the step cycle, which indicates the presence of plantar placement and weight support.^23^ The averaged hindpaw print area decreased by 4 wpi but significantly increased between 4 and 6 wpi (F_(1, 17)_ = 25.12, *p* = 0.0001); there were no significant differences between vehicle and ethanol groups (F_(1,17)_ = 0.01, *p* = 0.92, overall treatment effect; Fig. 3C). Base of support is the distance between left and right paws during one step cycle and stride length is the distance between consecutive steps of the same paw (Fig. 3A, top left). Hindpaw base of support decreased after injury but showed no significant difference between time (F_(1, 15)_ = 0.66, *p* = 0.43) or treatment (F_(1, 17)_ = 0.15, *p* = 0.70; Fig. 3D). Hindpaw stride length decreased after injury and between 4 and 6 wpi (F_(1,17)_ = 4.44, *p* = 0.05) but ethanol administration had no effect (F_(1,17)_ = 2.39, *p* = 0.14; Fig. 3E).

**FIG. 4.**
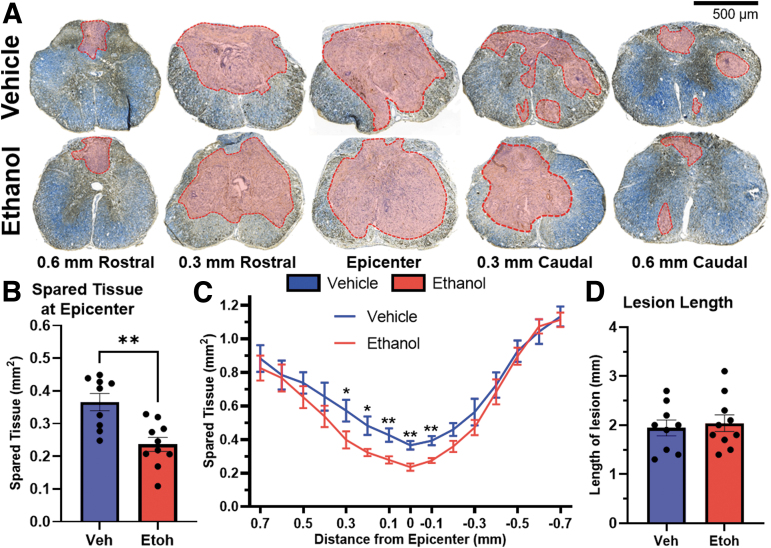
Acute ethanol intoxication significantly decreases spared tissue after SCI. **(A)** Representative images of the spinal cord throughout the rostral-caudal extent of injury 42 days after SCI. Sections are stained for myelin (blue, erichrome cyanine) and axons (brown, neurofilament). Frank lesion areas are highlighted in red. Spared tissue is evident by both blue and brown staining. Ethanol exposure at the time of SCI leads to less tissue sparing at the epicenter **(B)** and rostral to the epicenter **(C)**. Lesion length represents the total length of damaged (< 95% spared tissue) spinal cord tissue. Lesion length was not affected by ethanol exposure (**D**; B–D, Veh: *n* = 9, Etoh: *n* = 10); (B) unpaired *t*-test (*p < 0.05, **p < 0.01); (C) two-way repeated measures ANOVA with Fischer's least significant difference post hoc test (*p < 0.05). Error bars represent SEM. ANOVA, analysis of variance; Etoh, ethanol; SCI, spinal cord injury; SEM, standard error of the mean; Veh, vehicle.

To determine the long-term effects of acute ethanol exposure on secondary injury and anatomical recovery, we collected spinal cord tissue at 42 dpi. Representative images of spinal cord spanning 0.6 mm rostral and caudal to the injury epicenter are shown in Figure 4A. Frank tissue pathology (outlined in red) was identified as double negative regions that lack both myelin (eriochrome cyanine in blue) and axons (neurofilament in brown). Acute ethanol exposure at the time of injury leads to significantly less spared tissue at the lesion epicenter (*p* = 0.022; Fig. 4B) and rostral to the epicenter (Fig. 4C) relative to vehicle-treated controls (treatment effect: F_(1,17)_ = 2.672, *p* = 0.121). Lesion length is defined as the total distance of the spinal cord in which a maximum of 95% of the tissue is spared. Lesion length was not altered by acute ethanol intoxication (Fig. 4D).

## Discussion

In this article, we demonstrated that acute ethanol intoxication at the time of SCI exacerbates tissue loss and that isoflurane anesthetic can slow ethanol metabolism. The significant difference in lesion area observed in acutely intoxicated mice did not translate into worsened sensory or motor deficits. Ethanol consumption can be found in 30% of SCI cases and is a risk factor for more severe injuries.^24^ Therefore, our finding that ethanol intoxication at the time of injury can exacerbate lesion pathology is important to consider during the medical and surgical management of SCI patients with an elevated BAC.

Our findings corroborate clinical studies that report similar effects of elevated BAC on motor, sensory, and clinical outcomes following SCI. Furlan and colleagues report that elevated BAC at admission did not affect mortality, neurological impairment, or functional disability after SCI.^6^ Crutcher and associates likewise found no effect of BAC on mortality but also reported that an elevated BAC is associated with longer ICU stays and an increased risk for several complications.^4^ These findings highlight the importance of considering the systemic effects of ethanol when treating SCI in patients with an elevated BAC on admission. Our findings also support pre-clinical studies of feline and ferret models of SCI.^7–9^ These reports demonstrate that ethanol administration prior to SCI leads to more severe hemorrhage at the injury site,^8,9^ which may be a contributing factor to the exacerbated tissue pathology we report here.

We found that motor and sensory outcomes are not altered by ethanol intoxication, whereas a previous report in a rat SCI model found that ethanol intoxication at the time of injury causes a significant motor deficit.^7^ Mice metabolize ethanol 4 times faster than rats and 5 to 7 times faster than humans.^15,25^ The different metabolism rates may limit ethanol exposure after injury and limit ethanol's deleterious effects in mice compared with rats. Also, a retrospective clinical study reported that an elevated BAC at the time of admission was associated with improved motor recovery between rehabilitation admission and discharge.^5^ This study had a smaller sample size compared with the other reports and specifically focused on changes in motor function at later stages of recovery. Acute ethanol intoxication has varied effects on SCI outcomes in clinical and pre-clinical studies. Consequently, more investigations are needed to further clarify the effects of ethanol in SCI.

Acute ethanol intoxication is also common in the setting of traumatic brain injury (TBI). Approximately 30–60% of the patients admitted for TBI have an elevated BAC.^26^ Similar to SCI, it is unclear if ethanol intoxication is protective or detrimental to TBI outcomes. However, a meta-analysis of seven studies reports that ethanol intoxication leads to reduced in-hospital mortality in cases of severe TBI.^26^ A mouse model of TBI demonstrates a blunting effect of ethanol on neuroinflammation and immune cell activation along with a decrease in certain cytokines and improved neurological recovery.^27^ Other studies that used feline and rat TBI models report a harmful effect of ethanol with increased brain edema and tissue pathology.^9,28^ Lastly, a study conducted in rats reported a harmful effect of high-dose ethanol (3 g/kg) but a beneficial effect of a lower dose (1 g/kg) with regards to lesion volume and neurological impairment.^29^ In both TBI and SCI the effects of ethanol on injury outcomes are unclear and seem to be heavily dependent on species, administration route, and dose.

In other models of traumatic injury, such as burns and penetrating injuries, acute ethanol intoxication is associated with increased risk of infection and mortality.^30^ In rodents, acute ethanol intoxication alters wound healing by inhibiting neutrophil extravasation, leukocyte adhesion, and production of pro-inflammatory cytokines.^16,31,32^ In SCI, the role of neutrophils is complicated. Neutrophil manipulation is associated with enhanced recovery but also exacerbated damage.^33,34^ Therefore, our findings may be related to the lack of neutrophil extravasation acutely after injury while ethanol persists in circulation.

Our findings along with currently available clinical literature suggest that acute ethanol intoxication may not have a substantial impact on locomotor recovery after SCI. However, a patient's BAC at time of admission may have important ramifications for clinical decision-making in the emergent care setting. It is well established that at low BAC, ethanol is converted into acetaldehyde by alcohol dehydrogenase (ADH). At higher BAC ethanol is metabolized by both ADH and cytochrome P450 2E1 (CYP2E1).^35^ Interestingly, halogenated volatile anesthetics such as isoflurane are also metabolized by CYP2E1.^36^ Accordingly, our data show that isoflurane anesthesia slows ethanol metabolism. This finding is an important consideration during acute surgical interventions that occur immediately upon admission in SCI patients. If the patient's exposure to ethanol affects tissue pathology, anesthesiologists may want to opt for a drug that does not rely on the same clearance mechanism as ethanol.

The liver is primarily responsible for metabolizing ethanol and is innervated by autonomic and sensory fibers of the sympathetic and parasympathetic nervous systems that regulate its function.^37^ It is well established that SCI above vertebral level T6 in humans leads to autonomic dysfunction, which may alter liver function and lead to increased risk of cardiovascular and metabolic disease.^38–40^ To date, no studies have investigated if high-level-SCI-induced autonomic dysregulation affects ethanol metabolism.

One limitation of this study is the time-point assessed to determine tissue pathology and behavioral outcomes. Assessing intraspinal inflammation, at acute time-points (1, 3, and 7 dpi) may show altered pro-inflammatory microglia and macrophage activation, neutrophil extravasation, and hemorrhage.^41^ Altered neuroinflammation may be a consequence of the effects of ethanol above but also may be due to the excess edema associated with high doses of ethanol. Future studies should investigate the impact of ethanol exposure on neutrophil, microglia, and macrophage phenotype at earlier time-points. Additionally, therapeutics, such as tranexamic acid, which promote hemostasis, may reverse the excess edema induced by ethanol and have been shown to improve outcomes in mouse SCI.^42^ Additionally, the injury type, injury level, single sex, mouse strain, and single ethanol dose used in this study limit its external validity. SCI is very heterogeneous in age, sex, level, and modality in the human population. We have published reports that demonstrate the effect of sex on neuroinflammation, recovery, and pain development after SCI and do not consider this report to be sufficient to elucidate all interactions between SCI and ethanol intoxication.^21,43^

Lastly, C57BL/6J mice have a mutation in the nicotinamide nucleotide transhydrogenase (NNT) gene making them susceptible to oxidative stress. Both acute ethanol intoxication and SCI can deplete hepatic and intracellular stores of antioxidants such as glutathione.^44,45^ Therefore, it would be interesting to see if the same exacerbated tissue pathology is observed in other C57BL/6 strains that are not predisposed to oxidative stress. We employed one specific injury type, sex, species, strain, and age to interrogate the effects of an elevated BAC on SCI outcomes in a previously established T9 contusion model. As previously discussed, in other models of traumatic injury different doses of ethanol, administration routes, and species can have opposite effects on certain outcomes. Future studies investigating ethanol and SCI should employ other species, both sexes, different strains, and other injury models to tease out the subtle interactions between ethanol exposure and central nervous system (CNS) injury outcomes.

Another potential future direction is the use of chronic ethanol exposure to understand the interaction of heavy alcohol use on SCI outcomes. Individuals with SCI are 5 times more likely to have a history of heavy alcohol use prior to their injury compared with aged-matched controls.^46^ Additionally, chronic ethanol exposure has a myriad of harmful systemic effects that may alter SCI outcomes. Heavy alcohol use is associated with increased risk of bacterial and viral infection, cirrhosis, osteoporosis, and neurodegenerative diseases.^47^ These comorbidities could lead to worse SCI outcomes. Further, these future studies may identify potential therapeutic targets for individuals with a history of heavy alcohol use who suffer a SCI.

Our results demonstrate that ethanol intoxication prior to contusion SCI yields subtle differences in mice. Around 1 in 3 SCIs occur when individuals have an elevated BAC and several clinical and pre-clinical studies have interrogated the effect of ethanol on SCI outcomes with mixed results. Our findings corroborate the current literature, which shows that ethanol may have an incremental effect on outcomes after SCI. Additionally, we highlight a previously observed phenomenon of decreased ethanol metabolism in the presence of halogenated anesthetics. Overall, more studies should be conducted in other injury models that interrogate ethanol's effects on SCI outcomes to direct decision-making in this common clinical scenario.

## Transparency, Rigor, and Reproducibility Summary

*A priori* power analyses (1-β = 0.80; α = 0.05) were performed to determine the number of mice needed in each experiment. Groups of 10 mice are sufficient to detect differences (*p* < 0.05) between groups in locomotor recovery and tissue pathology using repeated measures ANOVA with post hoc comparisons. To account for attrition a total of 32 mice were injured (16 in each group). Of the 32 mice that were originally injured, 1 died during surgery, 5 were excluded for technical reasons, 1 was excluded for poor incision healing, and 1 was euthanized 1 day after SCI. Of the 8 mice that were excluded in behavioral analysis 4 were in the vehicle group and 4 were in the ethanol group. During analysis of tissue pathology, the first block of tissue was excluded due to poor tissue sectioning. This excluded 2 mice that were already excluded for technical reasons and 3 additional animals (2 in the vehicle group and 1 in the ethanol group). Ethanol administration occurred ∼40 min prior to SCI to achieve stable PECs at the time of injury. After baseline behavior was collected, all mice were randomized to either ethanol or vehicle using a random number generator. BMS scoring, Catwalk XT gait analysis testing, and Hargreaves heat sensitivity testing were all performed by observers blinded to the treatment group. Data from this study will be available in a FAIR data repository; contact a corresponding author for access information.
